# Severe Community-acquired Pneumonia Due to *Staphylococcus aureus*, 2003–04 Influenza Season

**DOI:** 10.3201/eid1206.051141

**Published:** 2006-06

**Authors:** Jeffrey C. Hageman, Timothy M. Uyeki, John S. Francis, Daniel B. Jernigan, J. Gary Wheeler, Carolyn B. Bridges, Stephen J. Barenkamp, Dawn M. Sievert, Arjun Srinivasan, Meg C. Doherty, Linda K. McDougal, George E. Killgore, Uri A. Lopatin, Rebecca Coffman, J. Kathryn MacDonald, Sigrid K. McAllister, Gregory E. Fosheim, Jean B. Patel, L. Clifford McDonald

**Affiliations:** * Centers for Disease Control and Prevention, Atlanta, Georgia, USA;; † Johns Hopkins Medical Institutions, Baltimore, Maryland, USA;; ‡ University of Arkansas for Medical Sciences College of Medicine, Little Rock, Arkansas, USA;; § Saint Louis University School of Medicine, Saint Louis, Missouri, USA;; ¶ Michigan Department of Community Health, Lansing, Michigan, USA;; # National Institutes of Health, Bethesda, Maryland, USA;; ** Oklahoma State Department of Health, Oklahoma City, Oklahoma, USA;; †† Washington State Department of Health, Shoreline, Washington, USA

**Keywords:** Staphylococcus, MRSA, pneumonia, influenza, research

## Abstract

During the 2003–04 influenza season, 17 cases of *Staphylococcus aureus* community-acquired pneumonia (CAP) were reported from 9 states; 15 (88%) were associated with methicillin-resistant *S. aureus* (MRSA). The median age of patients was 21 years; 5 (29%) had underlying diseases, and 4 (24%) had risk factors for MRSA. Twelve (71%) had laboratory evidence of influenza virus infection. All but 1 patient, who died on arrival, were hospitalized. Death occurred in 5 (4 with MRSA). *S. aureus* isolates were available from 13 (76%) patients (11 MRSA). Toxin genes were detected in all isolates; 11 (85%) had only genes for Panton-Valentine leukocidin. All isolates had community-associated pulsed-field gel electrophoresis patterns; all MRSA isolates had the staphylococcal cassette chromosome *mec* type IVa. In communities with a high prevalence of MRSA, empiric therapy of severe CAP during periods of high influenza activity should include consideration for MRSA.

## Abstract 2

*S. aureus* community-acquired pneumonia has been reported from 9 states.

*Staphylococcus aureus* is an infrequent cause of community-acquired pneumonia (CAP), accounting for ≈3% of cases in which a bacterial cause is identified, but it is a recognized cause of influenza-associated CAP (*1**–**4*). Methicillin-resistant *S. aureus* (MRSA) commonly causes nosocomial pneumonia, but relatively few cases of MRSA CAP have been reported (*5**,**6*).

Recent reports have shown that MRSA is an emerging cause of skin and soft tissue disease among otherwise healthy persons who have little or no contact with healthcare settings (*7**,**8*). These community-associated strains of MRSA differ from healthcare-associated strains by having a characteristic methicillin-resistant gene cassette (staphylococcal cassette chromosome *mec* [SCC*mec*] type IV) that elicits certain toxins, notably Panton-Valentine leukocidin (PVL), resistance generally limited to the β-lactams and macrolides, and specific molecular typing patterns (*8**–**10*).

During the 2003–04 influenza season, the Centers for Disease Control and Prevention (CDC) received reports of severe complications after influenza virus infection, including pneumonia caused by *S. aureus* and MRSA, among previously healthy children and adults. We report the demographic and clinical features of 17 patients with *S. aureus* and MRSA CAP associated with influenza or influenzalike illness (ILI) and describe the microbiologic characteristics of the *S. aureus* isolates.

## Methods

### Case Definition and Case Finding

A case of *S. aureus* CAP associated with ILI (*S. aureus* CAP-ILI) was defined as pneumonia occurring during the 2003–04 influenza season in a person with either laboratory-confirmed influenza virus infection, clinician-determined ILI (e.g., fever plus sore throat or cough), or both during the 2003–04 influenza season from whom a specimen (i.e., blood, sputum, or pleural fluid) collected <48 hours after hospitalization yielded *S. aureus*. Cases were identified by following up on reports of influenza-associated staphylococcal complications on 2 influenza assessment surveys conducted in December 2003 by the Infectious Diseases Society of America Emerging Infections Network, which consists of 859 infectious disease consultants (*11*). These surveys collected information on the 2003–04 influenza outbreak, including influenza-related complications, such as secondary bacterial infections, among pediatric and adult populations. Reports were also received through state and local health departments. Detailed clinical information on 4 cases was presented previously (*12*). We contacted clinicians and collected information by using a standardized data collection form on patient demographics, past medical history, signs and symptoms at the time the patient sought medical care, hospitalization, laboratory data including influenza testing, and risk factors for acquisition of MRSA (i.e., hospitalization, dialysis, surgery, or residence in a long-term care facility in the previous year; ever having an MRSA infection; and presence of percutaneous device or catheter at time of positive *S. aureus* culture). In addition, data on empiric (i.e., before *S. aureus* culture results were known) and targeted (i.e., after *S. aureus* culture results known) antimicrobial therapy and clinical outcomes were collected. Discordant empiric or targeted therapy was defined as a drug regimen that did not include an antimicrobial agent to which *S. aureus* was susceptible.

### Laboratory Procedures

*S. aureus* isolates from patients were collected and sent to CDC for characterization. All available isolates were tested for susceptibility to chloramphenicol, clindamycin, erythromycin, gentamicin, levofloxacin, linezolid, oxacillin, penicillin, rifampin, tetracycline, trimethoprim-sulfamethoxazole, and vancomycin by using broth microdilution, as recommended by the Clinical Laboratory Standards Institute (*13*). Inducible clindamycin resistance was determined for isolates with the erythromycin-resistant/clindamycin-susceptible phenotype by using the double-disk diffusion test (D-zone test) (*13*). All isolates were tested for genes encoding selected toxins (staphylococcal enterotoxin [SE] A–E, H; PVL; and toxic shock syndrome toxin 1) by multiplex real-time polymerase chain reaction (PCR) using primers prepared at CDC. All MRSA isolates underwent typing of their SCC*mec* gene cassette with PCR (*14*). Genotyping of all isolates was performed by pulsed-field gel electrophoresis (PFGE) with *Sma*I-digested DNA, and gels were analyzed as previously described (*9*).

## Results

### Case Characteristics

From November 10, 2003, to January 4, 2004, 17 cases of *S. aureus* CAP-ILI were reported from 9 states (Alabama, Arkansas, Illinois, Maryland, Michigan, Missouri, Oklahoma, Texas, and Washington); 15 (88%) were due to MRSA. The median age of the 17 case-patients was 21 years; 5 (29%) patients had underlying diseases, and 4 (24%) had risk factors for MRSA (Table 1) Although 5 (29%) patients were in the primary target groups (i.e., underlying illness [n = 2], age 50–64 years [n = 3]) recommended for annual influenza vaccination under current guidelines, only 1 (20%) had documented influenza vaccination during 2003–04. All case-patients had clinician-determined ILI. Twelve (71%) of the 17 patients had laboratory-confirmed influenza virus infection; 10 of these were confirmed by rapid antigen testing. *S. aureus* was recovered from multiple sources including sputum (14 [82%]), blood (8 [47%]), and pleural fluid (4 [24%].

**Table 1:** Demographic and clinical characteristics of cases of *Staphylococcus aureus* community-acquired pneumonia associated with influenzalike illness, influenza season 2003–04*

Characteristic	No. (%), N = 17
Median age, y (range)	21 (3 mo–62 y)
Sex, male	8 (47)
Race	
White	10 (59)
Black	7 (41)
Underlying disease†	5 (29)
MRSA risk factors‡	4 (24)
Documented influenza vaccination	1 (6)
Evidence of influenza infection	
Laboratory-confirmed	12 (71)§
Rapid antigen test	10 (59)
Paired serology	2 (12)
Fluorescent antibody staining	2 (12)
Clinical symptoms	
Cough	14 (82)
Myalgias	9 (53)
Sore throat	6 (35)
Headache	6 (35)
Shortness of breath	5 (29)
Rigors	4 (24)
Clinical signs	
Temperature >38°C	12/13 (92)
Hypotension (systolic blood pressure <90 mm Hg)	12/13 (93)
Normal or elevated leukocyte count† (>3,500/mm^3^)	12/16 (75)
Median leukocyte count (range)	16,500 mm^3^ (6,000–295,000)
Leukopenia (<3,500/mm^3^)	4/16 (25)
Thrombocytopenia (<150,000/mm^3^)	6/16 (38)
Radiologic documentation of pneumonia¶#	
Lobar	3/16 (19)
Multiple lobe involvement	4/16 (25)
Diffuse/patchy infiltrates	6/16 (38)
Effusions/empyema	5/16 (31)
Cavitation/necrosis	4/16 (25)

*MRSA, methicillin-resistant *S. aureus*. †One each of diabetes, multiple sclerosis, prune belly syndrome, cystic fibrosis, eczema. ‡Hospitalization, dialysis, surgery, or residence in a long-term care facility in the previous year; ever having an MRSA infection; and presence of percutaneous device or catheter at time of culture. §One patient had influenza infection confirmed by all methods. ¶One patient died on arrival at the hospital. #Not mutually exclusive.

Respiratory symptoms began a median of 4 days (range 1–17 days) before *S. aureus* specimen collection. All patients had >1of the following at the time they sought medical care: cough, myalgias, sore throat, headache, or shortness of breath. Most had fever, hypotension, and normal or elevated leukocyte counts. Four (25%) had leukopenia, and 6 (38%) had thrombocytopenia. Radiologic information was available for review for 16 patients, and all had documentation of an infiltrate. Information on empiric antimicrobial therapy was available for 15 patients; most received a third-generation cephalosporin (9 [60%]), respiratory fluoroquinolone (i.e., levofloxacin, gatifloxacin, or moxifloxacin) (7 [47%]), or vancomycin (10 [67%]); most patients (13 [87%]) received multiple antimicrobial agents. Discordant empiric antimicrobial therapy was documented in 3 (20%) patients, all of whom received a third-generation cephalosporin with or without a macrolide. Information on targeted antimicrobial therapy was provided for 13 patients (2 died before targeted treatment could be initiated) and consisted of vancomycin (10 [77%]), linezolid (2 [15%]), clindamycin (5 [38%]), and fluoroquinolones (4 [31%]); many patients (9 [69%]) received multiple antimicrobial drugs.

One patient was pronounced dead on arrival at the emergency department. Most patients were admitted to the intensive care unit and required intubation, and placement of chest tubes (Table 2). The median number of hospital days for patients was 13 (range 1–108 days). Five patients (4 with MRSA) died; their median age was 28 years (range 2 months–52 years), and only 1 had underlying illness (diabetes). Most died within 1 week of symptom onset.

**Table 2:** Outcomes of cases of *Staphylococcus aureus* community-acquired pneumonia associated with influenzalike illness, influenza season 2003–04

Outcome	No. (%), N = 17
Hospitalization	16 (94)*
Admitted to ICU†	13 (81)
Required intubation	8 (62)
Chest tube placement	6 (46)
Median length of stay (range)	13 days (1–108)
Death	5 (29)
Median age, y	28 (2–53)
Symptom onset to death, median days (range)	7 (3–73)
Underlying disease	1/5 (20)‡

*One patient died on arrival at the hospital. †ICU, Intensive care unit. ‡Diabetes.

### Laboratory Findings

*S. aureus* isolates were available from 13 (76%) patients (11 MRSA, 2 methicillin-susceptible *S. aureus*) from 9 states. Toxin genes were detected in all isolates; 11 (85%) had only the PVL genes, whereas 2 (15%) had genes for SEA, SEB, and SEH (Figure) All MRSA isolates had the SCC*mec* type IVa resistance gene cassette. Antimicrobial drug–susceptibility testing results for the MRSA isolates showed that all were resistant to oxacillin and erythromycin but susceptible to linezolid, rifampin, trimethoprim-sulfamethoxazole, and vancomycin; 10 (91%) and 6 (55%) isolates, respectively, were susceptible to clindamycin and levofloxacin. The 1 MRSA isolate that was not susceptible to clindamycin demonstrated inducible resistance by the D-zone test. In 4 cases, isolates were not available for testing at CDC. Antimicrobial drug susceptibility test results performed at the treating facility indicated that these 4 isolates were MRSA and nonsusceptible to macrolides (n = 4), clindamycin (n = 1), and levofloxacin (n = 1).

**Figure:**
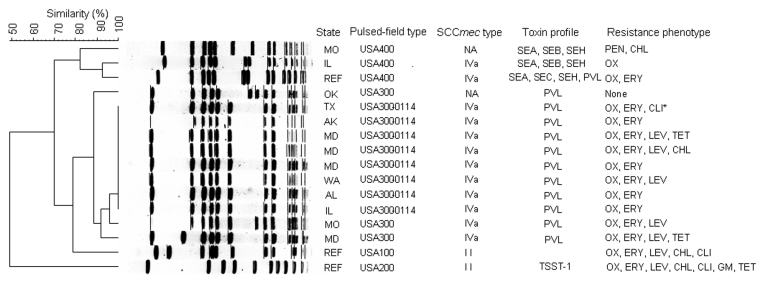
Dendrogram of *Staphylococcus aureus* isolates determined by using *Sma*I–digested DNA recovered from patients with community-acquired pneumonia associated with influenzalike illness, influenza season, 2003–04. NA, not applicable (methicillin-susceptible); SE, staphylococcal enterotoxin A, B, C, H; REF, reference strain; PVL, Panton-Valentine leukocidin; TSST, toxin shock syndrome toxin; CHL, chloramphenicol; CLI, clindamycin; ERY, erythromycin; GM, gentamicin; LEV, levofloxacin; OX, oxacillin; PEN, penicillin; TET, tetracycline. *Inducible clindamycin resistance.

Analysis of PFGE results showed that 11 (85%) were community-associated pulsed-field types USA300, and 2 (15%) were USA400, according to CDC criteria (Figure). Of the 10 MRSA isolates that were classified as pulsed-field type USA300, 8 (80%) from 6 different states had indistinguishable banding patterns and were further classified as USA300 subtype 0114. These MRSA isolates differed from pulsed-field types associated with healthcare-related strains (USA100 and 200) (*9*).

## Discussion

We report the emergence of *S. aureus* and MRSA as a cause of CAP-ILI resulting in severe illness and death in otherwise healthy persons in the United States during the 2003–04 influenza season. Most infections were caused by MRSA strains that contained PVL genes and were uniformly resistant to macrolides; half were nonsusceptible to fluoroquinolones. However, the isolates were susceptible to other antimicrobial agents, including vancomycin and linezolid. Although some phenotypic differences were noted, most cases of pneumonia appeared to be attributable to a single strain of MRSA found in diverse geographic areas. This strain, USA300 subtype 0114, is a predominant strain responsible for community outbreaks of MRSA skin disease in the United States (*8**,**9**,**15*).

Postinfluenza staphylococcal pneumonia has been reported in healthy adults during influenza pandemics and epidemics for the last century; it has been reported in the literature less frequently during the past 30 years (*1**–**3*). The recognition of MRSA as a cause of CAP-ILI has occurred concomitant with reports of MRSA as an increasingly common cause of skin and soft tissue infection in the community. Molecular typing of isolates in our series demonstrates that the CAP-ILI isolates are indistinguishable from MRSA associated with numerous outbreaks of skin and soft tissue infections (*8*). Given this association, MRSA might become a more common cause of *S. aureus* CAP following or coincident to influenza infection in regions where the MRSA strain is prevalent as a cause of skin and soft tissue infection. Antecedent *S. aureus* skin infection or colonization may be associated with postinfluenza *S. aureus* CAP, as was reported during the 1957 influenza pandemic (*16*). Although we did not systematically collect information on antecedent skin infections in our study, skin infections occurring among families of case-patients were noted. Given the apparent wide national distribution of MRSA as a cause of skin disease, physicians should be aware that MRSA can cause not only skin and soft tissue infections but also CAP.

Although most of the reported patients had laboratory confirmation of influenza virus as a cause of preceding illness, those diagnoses based solely on clinical symptoms may have been caused by other viral respiratory pathogens. However, growing evidence of mechanisms by which influenza may interact specifically with *S. aureus* to increase the risk for influenza–*S. aureus* co-infections suggests that these *S. aureus* CAP infections were likely associated with influenza (*17*). These include an influenza-induced increase in *S. aureus*–specific adhesion throughout the respiratory tract and *S. aureus*–specific proteases, which may increase influenza viral replication (*18**–**20*). This latter mechanism actually points to a synergistic relationship in which *S. aureus* increases influenza disease severity while influenza increases *S. aureus* infection and severity. Strains of influenza A virus also decrease phagocytic killing of *S. aureus*, leading to increased host susceptibility to bacterial superinfection (*21*). No other respiratory virus appears to share with influenza such a prominent role in predisposing to and increasing the severity of *S. aureus* pneumonia.

Risk factors for postinfluenza *S. aureus* CAP are undefined, but annual influenza vaccination is not recommended for half of the patients reported in our series under current guidelines (*22*). However, influenza vaccination is a major preventive strategy for influenza-associated pneumonia in older adults and in children 6–23 months of age (*22**,**23*). Moreover, studies have demonstrated that influenza vaccination can decrease the incidence of upper respiratory infections and lessen the need for antimicrobial drug use in healthy adults (*24**,**25*). Although these studies do not focus on specific bacterial complications, many studies have shown that influenza vaccination reduces overall pneumonia risk; thus one can reasonably assume that influenza vaccination would prevent secondary bacterial infections, including MRSA, in immunocompetent adults (*24**,**26*). Because information on antiviral treatment was not collected and most patients in this series sought medical care >2 days after illness onset, we could not assess the effects of early antiviral treatment. Although 1 study reported that early antiviral treatment of influenza with oseltamivir can decrease the incidence of lower respiratory tract complications, further studies are needed to determine whether early antiviral treatment of influenza can help reduce the risk for *S. aureus* pneumonia associated with influenza (*22**,**27*).

The incidence of MRSA CAP is unknown. In 2004, to monitor the incidence of MRSA, CDC initiated active population-based surveillance for invasive MRSA disease in 9 locations in the United States through the Emerging Infections Programs, Active Bacterial Core surveillance. These data will help characterize the further emergence of MRSA as a cause of CAP, guide public health interventions to prevent these infections, and provide information to guide empiric therapy recommendations. Currently recommended empiric therapy of CAP in immunocompetent adults with bacterial superinfection following influenza consists of a β-lactam or respiratory fluoroquinolone and may not adequately provide activity against community strains of MRSA (*28*). Whenever possible, physicians should obtain specimens (e.g., sputum or blood cultures) for diagnostic and antimicrobial drug–susceptibility testing to target therapy (*28**,**29*). Most patients in our series had severe disease and received broad-spectrum antimicrobial drugs, including coverage for resistant gram-positive bacteria. Whether initial inadequate empiric therapy plays a role in patient outcomes is therefore unknown.

Our cases suggest that empiric therapy of severe CAP during periods of high influenza activity should include coverage for MRSA, including among those without recognized risk factors for MRSA. In this regard, our concerns echo those of Martin et al. in 1959. Following these researchers' experience with the emergence of penicillin-resistant staphylococci during the 1957–58 Asian influenza pandemic, they commented "…during epidemics of influenza in localities in which staphylococci are known to be prevalent, all patients with signs of severe, potentially fatal influenza should—until proven otherwise—be diagnosed and treated promptly as cases of staphylococcal pneumonia caused by relatively antibiotic-resistant staphylococci" (*1*).
